# HOW DO OLDER ADULTS COPE WITH INTERSECTIONAL EXPERIENCES OF AGEISM AND RACISM?

**DOI:** 10.1093/geroni/igad104.1550

**Published:** 2023-12-21

**Authors:** Andrew Steward, Yating Zhu, Carson De Fries, Annie Zean Dunbar, Miguel Trujillo, Leslie Hasche

**Affiliations:** University of Wisconsin-Milwaukee, Milwaukee, Wisconsin, United States; University of Denver, Denver, Colorado, United States; University of Denver, Denver, Colorado, United States; University of Denver Graduate School of Social Work, Denver, Colorado, United States; Utah Division of Multicultural Affairs, Salt Lake City, Utah, United States; University of Denver, Denver, Colorado, United States

## Abstract

The World Health Organization recently launched a global campaign to end ageism due to its widespread, insidious negative health impacts on older adults. Similarly, racism is a key driver of health disparities and has been declared a public health crisis by jurisdictions around the United States. While a strong literature summarizes coping theory in general as well as beneficial and maladaptive coping mechanisms among older adults, limited research describes how older adults cope with experiences of ageism and racism, particularly through an intersectional lens. The aim of this qualitative, phenomenological study was to understand how older adults cope with intersectional experiences of ageism and racism. Twenty adults 60+ residing in the U.S. Mountain West who identified as Black, Hispanic/Latino(a), Asian-American/Pacific Islander, Indigenous, or White participated in a one-hour, semi-structured interview. A team of five coders engaged in an inductive coding process through independent coding followed by critical discussion. Peer debriefing, member checking, and an audit trail enhanced credibility. Nine themes were identified: 1) resistance, 2) exhaustion, 3) acceptance/‘let it go,’ 4) avoidance, 5) increased awareness through aging, 6) help others, 7) educate others, 8) self-determination/defy stereotypes, and 9) healthy lifestyle. These themes exemplify problem-focused and emotion-focused, as well as individual and collective coping strategies. Novel findings include how older adults may cope with ageism and racism through helping peer older adults and via increased awareness through aging. Practitioners should recommend coping strategies which combine collective and problem-focused responses to ageism and racism such as resistance, helping others, and educating others.

